# Artificial Intelligence and Heart-Brain Connections: A Narrative Review on Algorithms Utilization in Clinical Practice

**DOI:** 10.3390/healthcare12141380

**Published:** 2024-07-10

**Authors:** Giuseppe Micali, Francesco Corallo, Maria Pagano, Fabio Mauro Giambò, Antonio Duca, Piercataldo D’Aleo, Anna Anselmo, Alessia Bramanti, Marina Garofano, Emanuela Mazzon, Placido Bramanti, Irene Cappadona

**Affiliations:** 1 IRCCS Centro Neurolesi Bonino-Pulejo, Via Palermo, S.S. 113, C.da Casazza, 98124 Messina, Italy; giuseppe.micali@irccsme.it (G.M.);; 2 Department of Medicine, Surgery and Dentistry, University of Salerno, 84081 Baronissi, Italy; 3 Faculty of Psychology, Università degli Studi eCampus, Via Isimbardi 10, 22060 Novedrate, Italy

**Keywords:** artificial intelligence, machine learning, deep learning, algorithms, neurology, cardiology

## Abstract

Cardiovascular and neurological diseases are a major cause of mortality and morbidity worldwide. Such diseases require careful monitoring to effectively manage their progression. Artificial intelligence (AI) offers valuable tools for this purpose through its ability to analyse data and identify predictive patterns. This review evaluated the application of AI in cardiac and neurological diseases for their clinical impact on the general population. We reviewed studies on the application of AI in the neurological and cardiological fields. Our search was performed on the PubMed, Web of Science, Embase and Cochrane library databases. Of the initial 5862 studies, 23 studies met the inclusion criteria. The studies showed that the most commonly used algorithms in these clinical fields are Random Forest and Artificial Neural Network, followed by logistic regression and Support-Vector Machines. In addition, an ECG-AI algorithm based on convolutional neural networks has been developed and has been widely used in several studies for the detection of atrial fibrillation with good accuracy. AI has great potential to support physicians in interpretation, diagnosis, risk assessment and disease management.

## 1. Introduction

Cardiovascular disease (CVD) and neurological disorders (ND) are major causes of morbidity and mortality in both developed and poor countries and contribute significantly to reduced quality of life [1,2,3]. Due to the impact of these diseases on the global population, the use of artificial intelligence (AI) could be a valuable aid in the diagnosis, monitoring and treatment of diseases. Therefore, with this review we wish to analyse the state of the art of the application of AI in these clinical contexts. Recent studies report that neurological diseases were responsible for nearly 10 million deaths in 2019 [3], while cardiovascular diseases caused 18.6 million deaths worldwide in the same year [4]. According to the Global Burden of Diseases, Injuries, and Risk Factors study, communicable neurological disorders, stroke, headaches, neurodegenerative diseases, demyelinating diseases, brain and central nervous system tumours, and other less common neurological cancers are part of NDs [5]. CVMs, on the other hand, consist of ischemic heart disease, stroke, heart failure, peripheral arterial disease, and other cardiac and vascular diseases [6]. The most frequent risk factors for the occurrence of these chronic diseases are: diabetes mellitus, an unhealthy lifestyle, smoking, and age [7].

**Figure 1:**
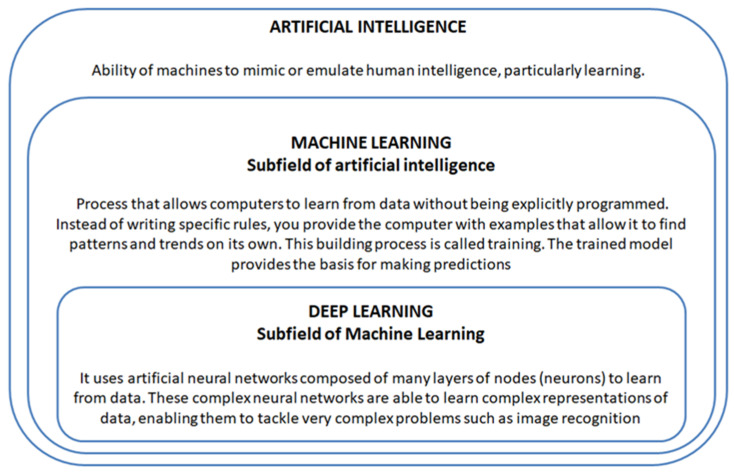
Artificial intelligence definition.

The heart–brain axis represents the complex interaction between the two systems and is important for maintaining cardiovascular and neurological health. The brain regulates the function of the heart, and altered brain function can cause cardiovascular disease. In turn, functional alterations in the heart can affect brain function [8]. Understanding this relationship is important for effective management of cardiovascular disease and neurological disorders. Cardiovascular disease (CVD) and neurological disorders (ND) are chronic conditions that require monitoring over time to effectively manage their progression and prevent complications. Artificial intelligence (AI) can play a significant role in supporting this monitoring process due to its ability to analyse large amounts of data and identify predictive patterns. AI is the ability of machines (computers) to mimic or emulate human intelligence, particularly learning, reasoning and planning [9]. Specifically, the computer receives data collected through various instrumentation, processes it and provides feedback. Machine Learning, Deep Learning, and neural networks are related concepts in the field of artificial intelligence [10]. Machine Learning is a subfield of Artificial Intelligence, Deep Learning a subfield of Machine Learning, and neural networks are the backbone of Deep Learning algorithms [11]. Machine Learning is a process that allows computers to learn from data without being explicitly programmed. Instead of writing specific rules, you provide the computer with examples that allow it to find patterns and trends on its own [12]. This construction process is called training. The trained model provides the basis for making predictions about new input values [13]. Machine learning is used in those areas where the design and programming of explicit algorithms with good performance is difficult or impractical. ML is also concerned with computational statistics, which focuses on the formulation of predictions using computers [14]. In detail, algorithm performance measures the ability of the model in question to solve a given type of problem. In classification problems, performance is estimated by the accuracy of the model and measured as the percentage of samples correctly predicted versus the error rate, i.e., the percentage of samples incorrectly predicted. Machine Learning algorithms can be categorized according to the type of experience they undergo during the learning process. There are: (i) supervised machine learning algorithms in which a pattern is built from labelled training data with the aim of devising a rule that associates matching input and output; (ii) unsupervised machine learning algorithms with patterns using unlabelled training data or without a matching output value, as the goal is to identify patterns in the inputs in order to be able to reproduce or predict them; (iii) machine learning algorithms with reinforcement through a system that can improve its performance through direct interactions with the environment [15]. Deep Learning uses artificial neural networks composed of many layers of nodes (neurons) to learn from data. These complex neural networks are able to learn intricate representations of data, enabling them to tackle very complex problems such as image recognition [16]. Neural networks can vary in complexity; however, the main components of neural networks are: (i) input layer that receives and processes the input signals by adapting them to the demands of the neurons in the network; (ii) one or more hidden layers that operate the processing (the number of layers depends on the performance of the algorithm), (iii) output layer that collects the results of the processing and adapts them to the demands of the next layer of the neural network [17]. Given the great potential of AI in the prediction and management of clinical data, its use is becoming increasingly widespread in support of medicine. Therefore, the purpose of our study is to assess the state of the art of AI application in cardiologic diseases and neurological disorders for their clinical impact on the general population. The concept definition of artificial intelligence and its domains is summarised in Figure 1.

## 2. Materials and Methods

### 2.1. Search Strategy

A descriptive review focusing on the analysis of studies on the application of artificial intelligence in health care in cardiology and neurology was conducted.

Studies were identified by consulting the literature databases PubMed, Embase, Web Of Science and Cochrane Library published before 20 November 2023. Articles from the past 10 years were considered. The keywords used for the search are (“artificial intelligence”[MeSH Terms] OR (“artificial”[All Fields] AND “intelligence”[All Fields]) OR “artificial intelligence”[All Fields] OR (“machine learning”[MeSH Terms] OR (“machine”[All Fields] AND “learning”[All Fields]) OR “machine learning”[All Fields]) OR “gpt”[All Fields]) AND (“cardiologi”[All Fields] OR “cardiologie”[All Fields] OR “cardiology”[MeSH Terms] OR “cardiology”[All Fields] OR “cardiology s”[All Fields]) AND (“neurology”[MeSH Terms] OR “neurology”[All Fields] OR “neurology s”[All Fields]).

The keyword search was conducted by one researcher and took about 3 days.

After removing duplicates, all articles were evaluated and selected based on title, abstract, and text. Evaluation of the studies using Microsoft Excel was completed over two rounds and each study was double-checked for inclusion by two researchers. This phase took about 2 months.

### 2.2. Inclusion Criteria

Studies meeting the following criteria were included in this review:

- Studies arguing both technical elements of artificial intelligence and clinical elements in cardiology and/or neurology
- Studies written in English;
- No Reviews;
- Studies published within the last 10 years;
- Studies that involved the use of clinical data.

### 2.3. Exclusion Criteria

A study was excluded if it lacked an analysis of the technical or clinical part. The restriction of the year of publication was adopted, excluding articles from before 2013. In addition, dissertations, a commentary, a letter or an editorial were excluded. Systematic, integrative or narrative reviews were also excluded, although their reference lists were checked and included if appropriate.

## 3. Results

**Figure 2:**
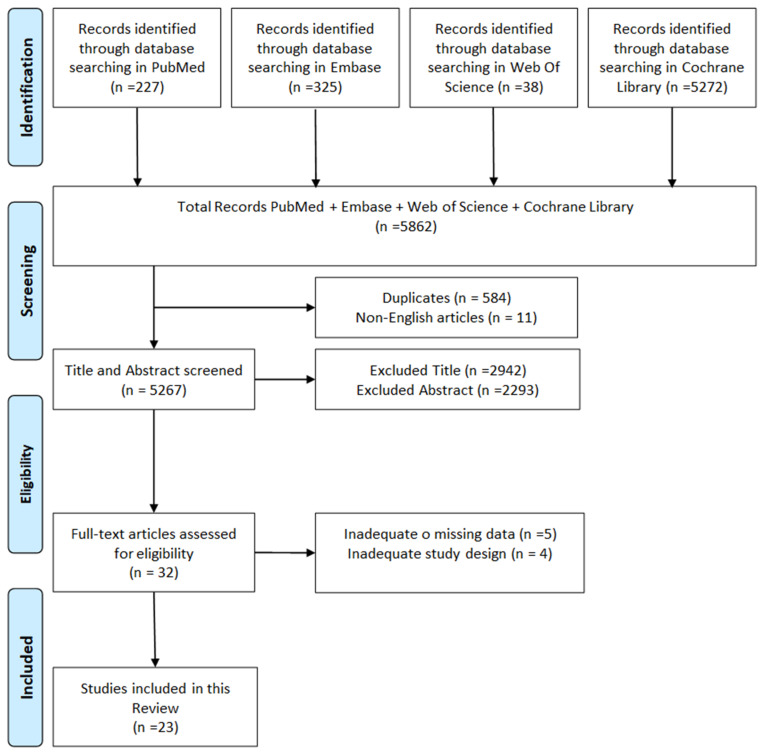
Graph of identification, screening, eligibility and inclusion of the review article.

Of the initial 5862 studies identified by our search, 23 met our inclusion criteria. See Figure 2.

Of the 23 studies, 14 had both Neurological and Cardiological scope as the study area, 7 purely Cardiological scope and 2 purely Neurological scope. See Table 1 for a detailed description of the studies.

### 3.1. Studies Conducted in Neurology and Cardiology

Andersson et al., 2021 [18] applied AI to prognosis in neurology and cardiology. In detail, they addressed the prediction of neurological outcome after OHCA using ANN with and without biomarkers. It analysed a sample of 932 patients via a temperature management study. The artificial intelligence algorithms considered are: RNAs with and without biomarkers, Bayesian algorithms and SHAP algorithms. The study observed that ANNs provide good to excellent prognostic accuracy in predicting the neurological outcome of post OHCA coma patients.

Johnsson et al., 2020 [19] applied AI to prognosis and prediction in Neurology and Cardiology. In detail, they studied the effects of intervention on severity classes in patients with cardiac arrest treated with TTM through an ANN-based model. It analysed a sample of 932 patients. The AI algorithm considered was an ANN-based supervised machine learning model. The study showed how the ANN model yielded better results than the logistic regression-based model and that ANN can be used to stratify a heterogeneous experimental population into risk classes and help determine the effects of intervention among subgroups.

Yoo et al., 2023 [20] applied AI to diagnosis in neurology and cardiology. In detail, they sought to develop an ECG deep learning algorithm capable of efficient screening of IPD. It analysed a sample of 1799 patients divided into three groups: 751 patients with IPD (experimental group), 751 non-IPD patients (control group), 297 patients with DPD. The artificial intelligence algorithm considered was a CNN-based deep learning algorithm. The study showed that the CNN-based deep learning model, using the 12-lead ECG, achieved relatively accurate performance in identifying patients with IPD.

Chiang et al., 2022 [21] applied AI to predictive in Neurology and Cardiology. In detail, they conducted an investigation of the probability of subclinical AF predicted by an AI-enabled algorithm on a single ECG in patients with MwA and MwoA. It analysed a sample of 40,002 patients (17,840 patients with MwA and 22,162 patients with MwoA). The AI algorithm considered was a CNN-based AI-ECG algorithm. The study, using a new AI-ECG algorithm, showed that patients with MwA have significantly higher AF prediction model output.

Chiu et al., 2022 [22] applied AI to prediction in neurology and cardiology. In detail, he identified predictors associated with TTM outcomes to predict survival and neurological outcomes of patients with ROSC treated with TTM. It analysed a sample of 580 patients with cardiac arrest and ROSC treated with TTM. The artificial intelligence algorithms considered are: ANN, LR and RF. The study showed that ANN techniques can accurately predict survival and neurological outcomes with higher sensitivity than LR and RF.

Sun et al., 2022 [23] applied AI to prediction in neurology and cardiology. In detail, they estimated the ability of rPPG measurement with DL models in discriminating AF from non-AF. It analysed a sample of 453 patients divided into three groups: 105 patients with AF (first group), 116 NSR patients (second group), 232 patients with abnormal ECG but normal AF (third group). Among the instrumentation used was a digital camera. The artificial intelligence algorithm considered was a DCNN algorithm. The study showed that the DCNN algorithm expressed 90% accuracy and 82% positive predictive value.

Zheng et al., 2022 [24] applied AI to predictive and monitoring in Neurology and Cardiology. In detail, they wanted to develop a DNN model to select AIS patients at high risk of post-stroke AF for prolonged cardiac monitoring and compare the model with other Machine Learning models. It analysed a sample of 3929 AIS patients. The AI algorithms that were considered are: LR, RFC, SVM, XGBoost and DNN. The study showed that The DNN model had the best specificity (94%), best positive predictive value (51%) and best accuracy (93%) compared with the other models in identifying patients with AIS at high risk of post-stroke atrial fibrillation.

Jamthikar et al., 2019 [25] applied AI to diagnosis in the Neurological and Cardiological fields. In detail, it provided an accurate and cost-effective ML-based system with stenosis as EEGS that can be used for routine CV/stroke risk assessment of patients. It analysed a sample of 202 patients. The AI algorithm that was considered was an RF. The study demonstrated that the delivered system resulted in an 18% improvement in the integrated ML approach compared to the conventional ML approach.

Kamel et al., 2020 [26] applied AI to prediction and diagnosis in the Neurological and Cardiological fields. Specifically, it trained a machine learning algorithm to distinguish cardioembolic strokes from non-cardioembolic strokes using data from the Cornell Acute Stroke Academic Registry. It analysed a sample of 1663 patients, of whom 1083 had a known aetiology of stroke, while 580 had a cryptogenic stroke defined according to the ESUS consensus definition based on information available at the time of the index hospitalization. The AI algorithms that were taken into consideration are: XGBoost, RF, LR and MARS. The study demonstrated that a machine learning algorithm trained on demographic, clinical, echocardiographic and laboratory data was able to distinguish known cardioembolic strokes from known non-cardioembolic strokes with excellent accuracy.

Hsiu et al., 2022 [27] applied AI to diagnosis in the neurological and cardiac fields. In detail, it sought to determine the effectiveness of using arterial pulse wave measurements, frequency domain pulse analysis, and machine learning analysis in distinguishing vascular aging. He analysed a sample of 100 patients. Among the instruments used, the use of a pressure transducer (KFG-2-120-D1-11, Kyowa, Nagoya, Japan) and a sphygmomanometer (MG150f, Rossmax International Ltd., Taipei, Taiwan) was envisaged. The AI algorithms that were taken into consideration are: MLP and RF. The study found significant differences in BPW spectral indices between vascular aging and control subjects. MLP and RF indicated that these indices can be used to facilitate the identification of vascular aging.

Mazza et al., 2022 [28] applied AI to prediction and monitoring in the Neurological and Cardiological fields. In detail, he developed a decision support tool to improve the management of extremely high blood pressure during the first 24 h after an acute ischemic stroke using machine learning (ML) tools. It analysed a sample of 7265 patients with acute ischemic stroke. The AI algorithms that were considered are: Decision Tree, RF, MLP and LR. The study demonstrated that the choice of antihypertensive treatment in the context of acute ischemic stroke should be adapted to the different blood pressure levels and clinical characteristics of the patient, thus providing a better decision-making approach. Furthermore, while ML techniques are useful for discovering hidden patterns in data and applying robust queries to datasets, they pose the risk of overfitting.

Shelly et al., 2023 [29] applied AI to prediction in the neurological and cardiac fields. In detail, it demonstrated premature aging in patients with mutations in the lamin A/C gene (LMNA) after hypothesizing that they have a biological age greater than their chronological age. He analysed a sample of 31 patients with LMNA. The AI algorithm that was taken into consideration was AI-ECG. The study demonstrated that AI-ECG predicted that patients with LMNA have a biological age greater than chronological age and accelerated aging even in the absence of cardiac abnormalities by traditional methods, and that raw ECG signals can identify the Chronological age and, importantly, differential aging rates in a genetic disease caused by mutations in LMNA.

Huang et al., 2023 [30] applied AI to prediction in the neurological and cardiac fields. In detail, he developed an IML model capable of accurately predicting 28-day all-cause mortality in hypertensive patients with ischemic or haemorrhagic stroke. It analysed a sample of 2526 hypertensive patients with ischemic or haemorrhagic stroke admitted to intensive care. The AI algorithms that were considered are: ANN, GBM, XGBoost, LR and SVM. The study demonstrated that the ML model that was developed has potential in clinical practice, as it can help personalize prevention and strengthen therapeutic strategies.

Iakunchykova et al., 2023 [31] applied AI to prognosis and prediction in the neurological and cardiac fields. In detail, it studied the effect of HDA on cognitive performance in a well-characterized Norwegian population cohort. Furthermore, she estimated BDA for a subgroup of participants to study the relationship between the two ML-based age estimators and its possible role as a mediator of the relationship between HDA and cognitive function. She analysed a sample of 7779 patients, of which 1694 had T1-weighted structural MRI available. Among the equipment used, the use of a Schiller AT104 PC [electrocardiogram (ECG)] was envisaged. The AI algorithm that was considered was a DNN. The study showed that heart delta age, which represents the cumulative effects of lifetime exposures, was associated with brain age. HDA was associated with cognitive function that was minimally explained through BDA.

### 3.2. Studies Conducted in the Field of Cardiology

Gruwez et al., 2023 [32] applied AI to diagnosis and monitoring in the field of Cardiology. Specifically, they aimed to determine the feasibility, detection rate, and therapeutic implications of large-scale screening for AF patients via smartphones. They analysed a sample of 60,629 patients. Among the equipment used, the use of a smartphone was planned. The AI algorithm considered is an algorithm integrated into a smartphone application based on PPG technology (FibriCheck©, Qompium, Hasselt, Belgium). The study demonstrated that AF screening based on smartphones is feasible on a large scale. Indeed, the quality of the photoplethysmography signal was sufficient for the analysis of 88% of the measurements. Furthermore, the algorithm classified the heart rhythm as normal in 80% of the measurements (without performing further actions), and as suspected AF in 10,231 (1.7%) measurements (confirmed after offline validation in 2998 measurements).

Khurshid et al., 2022 [33] applied AI to predictive cardiology. Specifically, they trained a convolutional neural network (“ECG-AI”) to predict the time to incidence of atrial fibrillation (AF). They analysed a sample of 45,770 patients. The AI algorithm considered was the ECG-AI algorithm. The study showed that ECG-AI can allow for efficient quantification of future risk of atrial fibrillation. Indeed, the algorithm achieved a reported positive predictive value of 92% for AF.

Fernandes et al., 2021 [34] applied AI to predictive cardiology. Specifically, they focused on improving the prediction of mortality after cardiac surgery by incorporating intraoperative risk factors into machine learning models. They analysed a sample of 5015 adult patients undergoing cardiac surgery. The AI algorithms considered were LR, RFC, ANN, SVM, and XGBoost. The study showed that machine learning models incorporating intraoperative adverse factors have the potential to provide better discriminative ability for risk stratification and patient triage after cardiac surgery. Indeed, all five models demonstrated good predictive ability (AUROC values ≥ 0.7). In particular, the XGB model showed better predictive ability, PPV, specificity, and sensitivity compared to the other models.

Debs et al., 2021 [35] applied AI to prognosis and prediction in the field of Cardiology. Specifically, they demonstrated the impact of integrating reperfusion status on the performance of deep learning models in predicting final infarction in patients with proximal intracranial occlusions treated with thrombectomy, comparing the results with current methods of clinical prediction. The study analysed a sample of 109 patients (74 patients with reperfusion and 35 patients without reperfusion). The AI algorithm considered is a CNN algorithm. The study showed that CNN-based models achieved higher DSC and AUC values compared to perfusion-diffusion mismatch models. CNN models achieved good accuracy, measured by a mean DSC of 78%.

Qutrio Baloch et al., 2020 [36] applied AI for prediction in the field of Cardiology. Specifically, they used machine learning to explore the relationship between functional limitation and symptom severity and severity of PAD. They analysed a sample of 703 patients with confirmed or suspected PAD. The AI algorithm considered in the study is a supervised ML algorithm composed of RF, NN, and GLM. The study demonstrated, through ML, that symptom severity assessed by the quality of life questionnaire is an important variable among patients with PAD. The algorithm achieved a positive predictive value of 66%, a specificity of 92%, and a sensitivity of 26%.

Wang et al., 2023 [37] applied artificial intelligence to predictive cardiology. Specifically, they conducted a study on genetic associations through estimates of atrial fibrillation risk generated by the ECG-AI model to evaluate the genetic basis reflected by the output. They analysed a sample of 39,986 patients. The AI algorithm considered is an ECG-AI algorithm. The study demonstrated that ECG-AI models can identify individuals at risk of disease through specific biological pathways. Indeed, estimating the genetic correlation between the AF risks predicted by ECG-AI and CHARGE-AF, a significant correlation of 39.3% was found.

Juarez-Orozco et al., 2020 [38] applied AI to diagnosis in the Cardiology field. In detail, it evaluated the feasibility and performance of ML using simple accessible clinical and functional variables for the identification of patients with ischemia or high risk of MACE determined through PET quantitative myocardial perfusion reserve (MPR). It analysed a sample of 1234 patients who underwent PET with nitrogen-13 ammonia. The AI algorithms that were taken into consideration are: LogitBoost, Naïve Bayes, RF and SVM. The study demonstrated that ML is feasible and applicable in the identification and use of simple and easily accessible predictors for the identification of patients who will have myocardial ischemia and an elevated risk of MACE as determined through quantitative imaging of the myocardial perfusion PET and that the implementation of ML in optimizing diagnostic technique selection, cardiovascular diagnosis, and prognostic estimates merits further research.

### 3.3. Studies Conducted in the Field of Neurology

Park et al., 2017 [39] applied AI to the diagnosis in the field of Neurology. Specifically, they developed a PDT tool with machine learning classifiers to detect stroke symptoms. They analysed a sample of 26 patients (16 stroke patients and 10 healthy patients). The instrumentation used included the use of a smartphone and two detection devices (placed on each of the subjects’ wrists). The AI algorithms considered were: SVM, RBFN, and RF. The study demonstrated that sensors and machine learning methods can reliably detect stroke signs and quantify proximal weakness of the arm. Indeed, it was shown that machine learning-based classifiers correctly classified up to 92.3% of PDT cases.

Amiri M et al., 2023 [40] applied AI to prediction in the Neurological field. In detail, it evaluated the accuracy of fMRI and EEG to identify residual consciousness in acute DoC in intensive care. It analysed a sample of 87 DoC patients, of which 51 were ≤UWS and 36 were ≥MCS. The AI algorithms that were taken into consideration are: Random Forest and SVM. The study demonstrated that by combining individual EEG- and fMRI-based models, an optimal combination of overall model performance, positive predictive value, and sensitivity can be achieved.

**Figure 3:**
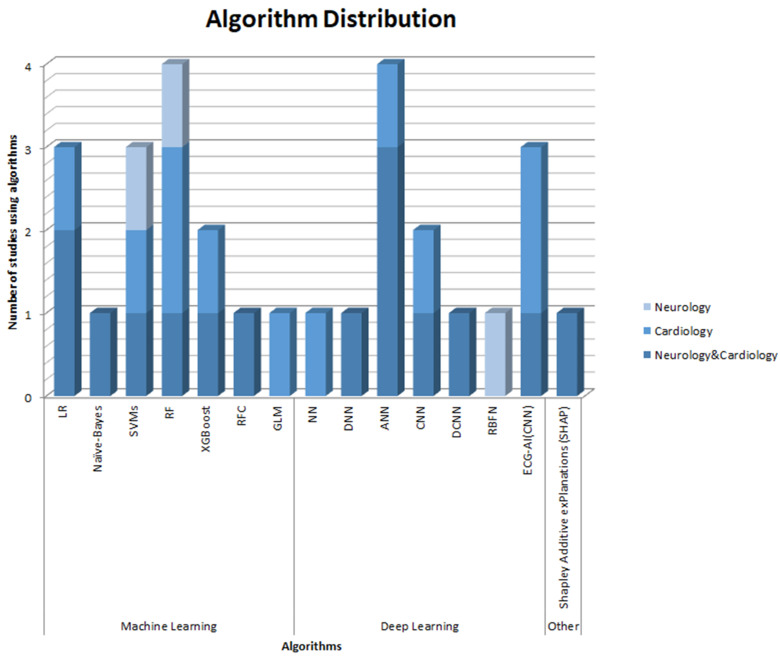
Algorithm distribution.

Overall, the most commonly used algorithms in these clinical areas are RF and ANN, followed by LR and SVMs. Additionally, a specific ECG-AI algorithm based on CNN has been developed. It has been widely used by various studies for detecting atrial fibrillation with good accuracy. The distribution of the use of individual algorithms is summarized in Figure 3.

**Figure 4:**
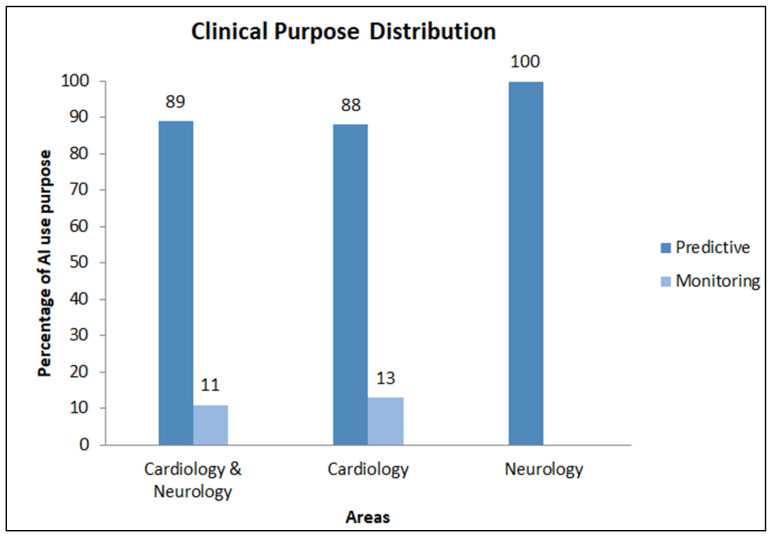
Purpose distribution by area.

The distribution of the purpose of use of AI algorithms indicates a clear prevalence of the predictive purpose, including the prognostic and diagnostic one, compared to that of monitoring aim. Figure 4 indicates the distribution of purpose in the studies included in this review divided by area.

## 4. Discussion

Artificial intelligence is becoming a very useful tool for guiding clinical decisions in many areas of medicine, including orthopaedics, oncology, and chronic inflammation [41,42,43]. Our review specifically explored the use of artificial intelligence algorithms in departments with higher rates of acute events, such as neurology and/or cardiology. Acute events like stroke and myocardial infarction are major causes of mortality and morbidity with significant clinical and socioeconomic impact [44]. Therefore, analysing the state of the art on the use of artificial intelligence algorithms in these areas can be crucial in predicting the risk of pathology, diagnosing, or predicting the prognosis of such disorders [45]. The main purpose of applying AI in these areas is to predict the onset of disorders or their progression over time [46].

Among the studies included in our review, it emerged that only one article focused exclusively on neurological aspects, while the majority of articles exclusively addressed cardiological aspects or analysed the interconnection between neurology and cardiology. This indicates an increasing connection between the two areas from the perspective of the so-called heart–brain axis [47]. The algorithms most commonly used in the studies included in this review are RF and ANN, followed by LR and SVMs.

In particular, the Random Forest (RF) algorithm has been utilized in all clinical fields. The RF is one of the most commonly used supervised Machine Learning algorithms for solving classification and regression problems [48]. It is an ensemble learning method that is based on constructing many decision trees during the training phase. Ensemble methods achieve qualitatively better predictive performance by using multiple learning algorithms. Decision trees create random forests using bagging or bootstrap aggregating methods. This is a method used to reduce the variance of an estimated prediction function and appears to be effective with high variance and low bias procedures.

The Artificial Neural Network (ANN) [49] is a computational network that functions similarly to the human brain, with interconnected neurons in various layers of the network. The depth of these interconnections is the basis of the concept of Deep Learning, to which ANN belongs, along with other algorithms. ANN solves problems that would be impossible or difficult by human or statistical standards. ANN can be considered a piece of computer system design intended to simulate the way the human brain analyses and processes information. ANN is further subdivided into three classes known as MultiLayer Perceptron (MLP), Convolutional Neural Network (CNN) and Recurrent Neural Network (RNN). Indeed, Deep Learning is represented by learning models inspired by the structure and functioning of the human brain, synthesized in the input-processing-output paradigm based on the use of Deep Neural Networks (NN), also known as Multilayer Neural Networks. ANN receives the input signal from an external source in the form of patterns and the image in the form of a vector. Depending on the path of the signal, we distinguish between Feedback ANN and Feedforward ANN. In Feedback ANN, the output cyclically returns within the network to achieve more evolved results and is specialized in solving optimization problems. On the contrary, Feedforward ANN allows information to flow unidirectionally from input nodes to output nodes through hidden layers, without cycles within the network.

Logistic Regression (LR) [50] is one of the most widely used Machine Learning algorithms for binary classification, when the dependent variable is dichotomous. During the training phase, the algorithm, belonging to the eager learner type (slower in the learning phase but faster in the prediction test phase), processes a distribution of weights. The goal of logistic regression is to model the weights to maximize the probability and minimize the error according to the statistical likelihood function of the model. At the end of training, the algorithm produces a model, which can be used to classify any other example not included in the training set. The main advantages of logistic regression are ease of use, interpretability, and scalability.

Support Vector Machines (SVMs) [51] are supervised learning models associated with learning algorithms for regression and classification. The algorithm is based on representing points in space where the line separating the classes is found. The calculation of the line is performed using only a portion of the dataset, namely the values of a class that are closest to the separating line, called “support vectors”. The goal of the algorithm is therefore to find a plane that maximizes the margin, the distance between the points of the two classes. During the training phase, the SVM algorithm calculates the line, while in the testing phase, it categorizes new input data by placing them in the part of the plane of the identified class.

In addition, a specific ECG-AI algorithm has been developed [52] based on CNN found in studies exclusively focused on cardiology and those investigating the correlation between neurological and cardiovascular aspects. This algorithm uses convolutional neural networks (CNN) in deep learning to examine the information provided by single, continuous, and intermittent ECG leads. It allows for the automated interpretation of the ECG. CNNs detect numerous visual properties as they are fed pixel values, and they provide superior performance with image, voice, or audio signal inputs. CNNs are characterized by three main types of layers: (i) the convolutional layer performs most of the computation and is composed of input data, filter and feature map, (ii) pooling layer reduces dimensionality through pooling processes; (iii) fully connected layer connects the pixel values processed from the input image to the output layer [53]. These artificial intelligence algorithms represent effective and non-invasive biomarkers for cardiovascular diseases. This also enables the identification of subtle signals in the ECG that may not be apparent to the human eye. The interpretation of atypical ECG traces also helps resolve old and new diagnostic problems [54,55]. One of the most recognized advantages of artificial intelligence in ECG analysis is the rapid and accurate detection of issues such as arrhythmias, silent heart diseases, and left ventricular failure. The growing interest in the use of artificial intelligence in various clinical fields could help develop algorithms useful in monitoring chronic pathologies in the neurological and cardiological areas, which often occur simultaneously and influence each other.

The main strengths and weaknesses of the algorithms analysed in this review are summarised in Table 2.

In addition, since chronic pathologies are increasingly widespread, monitoring would facilitate continuous but sustainable management for the patient, improving their quality of life. The use of AI is changing the way cardiac and neurological conditions are assessed, diagnosed and managed, leading to significant improvements in clinical outcomes and patient care. Timely analysis of images, clinical and biological data using AI has proven to be able to accurately diagnose various pathological conditions. This can lead to faster and more accurate diagnosis by enabling timely interventions even in rural areas or distant from hospitals and improving treatment perspectives for patients suffering from these conditions. Indeed, in addition to chronic neurological and cardiological conditions, potentially related conditions such as cognitive impairment, reduced exercise capacity, which can be assessed, monitored and treated remotely, must also be adequately considered [56].

This study has the strength of analysing the application of artificial intelligence algorithms in neurology and cardiology contexts, which are two areas of high interest due to the prevalence of these pathologies in the global population and their related clinical and socioeconomic implications. This presents a challenge to the application of artificial intelligence algorithms due to the need to train them to identify potential intrinsic physiopathological differences in various populations [57]. At the same time, the socioeconomic differences between countries do not allow for a uniform application from a geographical point of view [58]. However, this study also has limitations. Firstly, it is based on a limited number of studies. Additionally, the included studies have adopted different approaches. Another challenge faced in the writing of this study concerns the multidisciplinary integration necessary to address this topic [59]. Indeed, not all clinicians have the necessary computer skills to address the topic, which can limit doctors’ confidence in the use of artificial intelligence [60]. On the other hand, computer experts may not be familiar with various clinical fields. Therefore, integrating artificial intelligence tools into existing healthcare systems can be complex and require close collaboration between health professionals and informatics experts. An important resource is also the application of hybrid algorithms, particularly those inspired by nature that mimic biological and ecological processes. By being able to address the complex and dynamic nature of healthcare data in Internet of Things (IoT) environments, they can improve the efficiency, accuracy and adaptability of healthcare systems [61]. Addressing these aspects in training programs and implementing the application of artificial intelligence algorithms in various healthcare fields can support clinical practice more and more. The implementation of AI algorithms in clinical settings presents additional challenges that need to be addressed to increase the effectiveness of such tools [62]. One of the main challenges is data privacy. The use of artificial intelligence algorithms requires the collection and analysis of large amounts of personal patient data. It is essential for the healthcare system to ensure the protection and secure use of personal data in conformity with current regulations. Another condition to focus on is the need for large datasets for algorithm training. In clinical settings, it can be difficult to find and access adequately sized datasets, especially considering the sensitivity of patients’ personal data and the pathophysiological variability of each individual.

## 5. Future Perspectives

Further research in this direction could lead to the development of more accurate AI algorithms for the early diagnosis and monitoring of cardiac and neurological diseases in the future. This could facilitate the creation of predictive models capable of identifying early signs of heart and neurological diseases, enabling earlier preventive and curative interventions. Furthermore, considering the variability of each individual and the need to personalise treatments, machine learning algorithms could be developed that are able to predict individual response to treatments and optimise therapy according to specific patient characteristics, under the constant supervision of the physician. Indeed, communication between healthcare professionals and patients remains a key element in the treatment process [63]. Technological advancement in clinical areas offers numerous opportunities to improve the management of increasingly prevalent chronic diseases, but the harmonious development of new methods requires active cooperation between clinicians and informaticians. Only active collaboration can lead to the development of effective predictive models and machine learning algorithms specific to cardiac and neurological diseases through the appropriate collection and analysis of large amounts of clinical, genomic and imaging data to train and validate artificial intelligence models. In conclusion, research should continue to evaluate the clinical impact of new artificial intelligence technologies through randomised controlled trials and meta-analyses to ensure their efficacy and safety in clinical practice.

## 6. Conclusions

The application of artificial intelligence algorithms has a great potential to support doctors in interpretation, diagnosis, risk assessment, and disease management. However, it requires constant attention to datasets, continuous validation among populations, and targeted strategies to mitigate biases. In the future, greater application of AI algorithms in the monitoring of cardiological and neurological pathologies could facilitate the remote control of increasingly widespread pathologies in the population, reducing healthcare costs and improving the quality of life of patients.

## Figures and Tables

**Table 1:** Summary of studies included in the research.

Area	References	Aim	Sample (N)	Observation Area	Devices Used	AI Algorithm	Results
Neurology and Cardiology	Andersson et al., 2021 [18]	Predicting neurological outcome post OHCA using ANN with and without biomarkers	932 patients By target temperature management study	Prognosis	Missing.	ANN with and without biomarkers; Bayesian algorithms; SHAP algorithms	ANN provide good-to-excellent prognostic accuracy in predicting neurological outcome in comatose patients post OHCA, with an AUROC between 82% and 94% depending on the variables.
	Johnsson et al., 2020 [19]	Studying the effects of the intervention on the severity classes in patients with cardiac arrest treated with TTM, through a model based on ANN.	932 patients	Prognosis Predictive	Missing.	Supervised machine learning model based on ANN.	The ANN model has produced better results compared to the logistic regression model. The ANN can be used to stratify a heterogeneous experimental population into risk classes and help determine the effects of intervention among subgroups (AUC 0.852).
	Yoo et al., 2023 [20]	Develop a deep learning algorithm for ECG capable of efficiently screening for IPD	751 patients IPD (experimental group); 751 patients without IPD (control group); 297 patients with DPD.	Diagnosis	Missing.	Deep Learning algorithm based on CNN.	The CNN-based deep learning model using 12-lead ECG had relatively accurate performance in identifying patients with IPD (AUC 0.85).
	Chiang et al., 2022 [21]	Investigating the probability of subclinical AF predicted by an artificial intelligence-enabled algorithm on a single ECG in patients with MwA and MwoA.	17,840 patients MwA; 22,162 patients MwoA	Predictive	Missing.	AI-ECG based on a CNN	Using a new AI-ECG algorithm, it has been shown that patients with MwA have a significantly higher prediction model output of AF, with an accuracy of 83.3%.
	Chiu et al., 2022 [22]	Identifying predictors associated with outcomes for targeted temperature management (TTM) to predict survival and neurological outcomes for patients with ROSC treated with TTM.	580 patients with cardiac arrest and ROSC treated with TTM.	Predictive	Missing.	ANN (AUC 0.841); LR; RF.	ANN techniques can accurately predict survival and neurological outcomes with higher sensitivity compared to LR and RF (ANN sensitivity 71.6; LR sensitivity 40.5, RF sensitivity 45.3).
	Sun et al., 2022 [23]	Estimating the measurement capacity of rPPG with DL models in discriminating AF from non-AF	105 patients with AF (first group); 116 patients NSR (second group); 232 patients with abnormal ECG but normal AF (third group).	Predictive	Digital camera	DCNN	The algorithm has demonstrated an accuracy of 90% and a positive predictive value of 82%.
	Zheng et al., 2022 [24]	Developing a DNN model to select AIS patients at high risk of post-stroke AF for prolonged cardiac monitoring and comparing the model with other Machine Learning models.	3929 patients affected by AIS	Predictive Monitoring	Missing.	5 models of Machine Learning: LR; RFC; SVM; XGBoost; DNN.	The DNN model achieved the highest specificity (94%), the highest positive predictive value (51%), and the highest accuracy (93%) compared to other models in identifying patients with AIS at high risk of post-stroke atrial fibrillation.
	Jamthikar et al., 2019 [25]	Develop an accurate and cost-effective ML-based system with stenosis such as EEGS that can be used for routine CV/stroke risk assessment of patients	202 patients	Diagnosis	Missing	RF.	The system demonstrated an 18% improvement in the ML approach integrated with clinical data such as stenosis compared to the conventional ML approach (AUC = 0.68).
	Kamel et al., 2020 [26]	Training a machine learning algorithm to distinguish cardioembolic from non-cardioembolic strokes using data from the Cornell Acute Stroke Academic Registry.	1663 patients: 1083 with known stroke aetiology, 580 with cryptogenic stroke	Diagnosis Predictive	Missing	XGBoost; RF; LR; MARS.	A machine learning algorithm trained on demographic, clinical, echocardiographic, and laboratory data was able to distinguish known cardioembolic strokes from known non-cardioembolic strokes with excellent accuracy (85%).
	Hsiu et al., 2022 [27]	To determine the effectiveness of using arterial pulse wave measurements, frequency domain pulse analysis, and machine learning analysis in distinguishing vascular aging.	100 patients	Diagnosis	Pressure transducer (KFG-2-120-D1-11, Kyowa); Sphygmomanometer (MG150f, Rossmax)	MLP; RF.	This study found significant differences in BPW spectral indices between vascular aging and control subjects. MLP and RF are useful for identifying vascular aging through these indices, with accuracy >80%.
	Mazza et al., 2022 [28]	Development of a decision support tool to improve the management of extremely high blood pressure during the first 24 h after acute ischemic stroke using ML tools.	7265 patients with acute ischemic stroke.	Predictive Monitoring	Missing	Decision tree; RF; MLP; LR.	Antihypertensive treatment in the context of acute ischemic stroke should be adapted to different blood pressure levels and clinical characteristics of the patient, thus providing a better decision-making approach. ML techniques are useful for discovering hidden patterns from data (accuracy 97%) and applying robust queries to datasets, but they pose the risk of overfitting.
	Shelly et al., 2023 [29]	Demonstrate premature aging in patients with lamin A/C (LMNA) gene mutations after hypothesizing that they have a biological age greater than chronological age	31 LMNA patients	Predictive	Missing	AI-ECG	AI-ECG predicted that patients with LMNA have a biological age greater than the chronological age and accelerated aging even in the absence of cardiac abnormalities by traditional methods. Raw ECG signals can identify chronological age and, importantly, differential aging rates in a genetic disease caused by mutations in LMNA.
	Huang et al., 2023 [30]	Development of an IML model that can accurately predict 28-day all-cause mortality in hypertensive patients with ischemic or haemorrhagic stroke.	2526 hypertensive patients with ischemic or haemorrhagic stroke admitted to intensive care.	Predictive	Missing	ANN; GBM; XGBoost; LR; SVM.	The IML model developed in this study has potential in clinical practice, as it can help personalize prevention and strengthen therapeutic strategies (mean AUC value 75%).
	Iakunchykova et al., 2023 [31]	To study the effect of HDA on cognitive performance and estimation of BDA for a subgroup of participants through MRI imaging to study the relationship between the two ML-based age estimators and its possible role as a mediator of the relationship between HDA and cognitive function.	7779 participants by a Norwegian cohort: of which 1694 had T1-weighted structural MRI available.	Prognosis Predictive	Schiller AT104 PC [ECG]	DNN	Delta cardiac age, which represents the cumulative effects of lifetime exposures, was associated with brain age. HDA was associated with cognitive function that was minimally explained through BDA.
Cardiology	Gruwez et al., 2023 [32]	To determine the feasibility, detection rate, and therapeutic implications of a large-scale screening in AF patients through smartphones.	60,629 patients	Diagnosis Monitoring	Smartphone.	Smartphone application based on PPG technology (FibriCheck©, Qompium, Hasselt, Belgium).	AF screening based on smartphones is feasible on a large scale. Indeed, the quality of the photoplethysmography signal was sufficient for the analysis of 88% of the measurements. Furthermore, the algorithm classified the heart rate as normal in 80% of the measurements (without taking further actions), and as suspected AF in 10,231 (1.7%) measurements (confirmed after offline validation in 2998 measurements).
	Khurshid et al., 2022 [33]	Training of a convolutional neural network (“ECG-AI”) to explicitly predict the time to the onset of atrial fibrillation.	45,770 patients	Predictive	Missing.	ECG-AI.	The ECG-AI can allow for an efficient quantification of future risk of atrial fibrillation. Indeed, the algorithm has achieved a reported positive predictive value of 92% for AF.
	Fernandes et al., 2021 [34]	Improvement in mortality prediction accuracy after cardiac surgery by machine learning models incorporating intraoperative risk factors.	5015 adult patients undergoing cardiac surgery	Predictive	Missing.	LR; RF; ANN; SVM; XGBoost.	Machine learning models that incorporate intraoperative adverse factors have the potential to provide better discriminative ability for risk stratification and patient triage after cardiac surgery. Indeed, all five models demonstrated good predictive ability (AUROC values ≥ 0.7). In particular, the XGB model showed better predictive ability, PPV, specificity, and sensitivity compared to the other models.
	Debs et al., 2021 [35]	Impact of reperfusion state integration on the performance of deep learning models in predicting final infarct in patients with proximal intracranial occlusions treated with thrombectomy, and comparison of results with current clinical prediction methods.	74 patients with reperfusion; 35 patients without reperfusion	Prognosis Predictive	Missing.	CNN.	CNN-based models achieved higher DSC and AUC values compared to those of the perfusion-diffusion mismatch models. The CNN models achieved good accuracy, measured with an average DSC of 78%.
	Qutrio Baloch et al., 2020 [36]	Using machine learning to explore the relationship between functional limitation and symptom severity and severity of PAD.	703 patients with confirmed or suspected PAD	Predictive	Missing.	Supervised ML composed of RF, NN, and GLM	ML has shown that the severity of symptoms evaluated by the quality of life questionnaire is a highly important variable among patients with PAD. The algorithm achieved a positive predictive value of 66%, a specificity of 92%, and a sensitivity of 26%.
	Wang et al., 2023 [37]	Study on genetic association through atrial fibrillation risk estimates generated by the ECG-AI model to evaluate the genetic basis reflected by the output.	39,986 patients	Predictive	Missing	ECG-AI	ECG-AI models can identify individuals at risk of disease through specific biological pathways. Indeed, by estimating the genetic correlation between the risks of AF predicted by ECG-AI and CHARGE-AF, a significant correlation of 39.3% was found.
	Juarez-Orozco et al., 2020 [38]	To evaluate the feasibility and performance of ML in identifying patients with ischemia or high risk of MACE determined through quantitative myocardial perfusion reserve (MPR) PET	1234 patients underwent PET with nitrogen-13 ammonia	Diagnosis	Missing	LogitBoost algorithms; Naïve Bayes algorithms; RF; SVM.	ML is feasible and applicable to identify patients who will have myocardial ischemia and an elevated risk of MACE through simple predictors and quantitative myocardial perfusion PET imaging (AUC 0.71).
Neurology	Park et al., 2017 [39]	Development of a PDT tool with machine learning classifiers to detect stroke symptoms	16 patients with Stroke; 10 healthy patients	Diagnosis	Smartphone; 2 detection devices (positioned on each subject’s wrists)	SVM; RBFN; RF.	Sensors and machine learning methods can reliably detect signs of stroke and quantify proximal arm weakness. Indeed, machine learning-based classifiers have been shown to correctly classify up to 92.3% of PDT cases.
	Amiri et al., 2023 [40]	To evaluate the accuracy of fMRI and EEG to identify residual consciousness in acute DoC in the ICU.	87 DoC patients: 51 with UWS and 36 with MCS.	Predictive	Missing	RF; SVM.	By combining individual EEG- and fMRI-based models, an optimal combination of overall model performance, positive predictive value, and sensitivity can be achieved (AUC 79%).

Legend: AF = Atrial Fibrillation; AI = Artificial Intelligence; AI-ECG = ECG algorithm; AI-ECG = ECG algorithm enabled by artificial intelligence; AIS = Acute Ischemic Stroke; ANN = Artificial Neural Networks; AUC = Area Under the Curve; BDA = Brain delta age; BP = Blood Pressure; BPW = blood-pressure waveform; CNN = Convolutional Neural Network; DCNN = Deep Convolutional Neural Network; DL = Deep Learning; DNN = Deep Neural Networks; DoC = Disorders of consciousness; DPD = Drug-induced Parkinson’s Disease; DSC = Dice Similarity Coefficient; ECG = Electrocardiogram; EEGS = event-equivalence gold standard; fMRI = Functional MRI; GBM = Gradient boosting machine; GLM = Generalized Linear Model; HDA = Heart delta age; IML = Interpretable machine learning; ICU= Intensive Care Unit; IPD = Idiopathic Parkinson’s Disease; LMNA = Mutations in the gene for lamin A/C; LR = Logistic Regression; MACE = Major adverse cardiovascular events; MARS = Multivariate adaptive regression splines; MCS = minimally conscious state; MLP = feed-forward multi-layer perceptron (neural network); MPR = myocardial perfusion reserve; MRI = Magnetic Resonance Imaging; MwA = Migraine With Aura; MwoA = Migraine Without Aura; NN = Neural Network; NSR = Normal Sinus Rhythm; OHCA = Out of Hospital Cardiac Arrest; PDT = Pronator Drift Test; PET = positron emission tomography imaging; PPV = Positive Predictive Value; RBFN = Radial Basis Function Network; RF = Random Forests; RFC = Random Forest Classifier; ROSC = Return Of Spontaneous Circulation; rPPG = Remote Photoplethysmography imaging; SHAP = Shapley Additive exPlanations; SVM = Support Vector Machine; TIA = Transient Ischemic Attack; TTM = Targeted Temperature Management; UWS = unresponsive wakefulness state; XGBoost = Extreme Gradient Boosting.

**Table 2:** Strengths and weaknesses of the algorithms.

Algorithms	Strengths	Weaknesses
Random Forest (RF)	– High accuracy through ensemble of multiple decision trees; – Robust to noise and handles outliers well; – Non-parametric, flexible for various datasets; – Estimates feature importance for better model understanding; – Handles missing data and both numeric and categorical features.	– Computationally and memory-intensive; – Longer prediction times; – Less interpretable (black-box nature); – Risk of overfitting.
Logistic Regression (LR)	– Easy to implement and interpret, efficient training; – Assumes no specific distribution of classes; – Extensible to multinomial regression and probabilistic predictions; – Provides clear coefficient importance and direction; – Fast classification and generally good accuracy for simple datasets.	– Assumes linearity between variables; – Not suitable for datasets with more features than observations; – Constructs linear decision boundaries, not ideal for nonlinear problems; – Requires no multicollinearity among features; – Difficult to capture complex relationships.
Support-Vector Machines (SVMs)	– Effective for high-dimensional and small datasets; – Models nonlinear boundaries with kernel trick; – Robust to noise and has good generalization; – Versatile for classification and regression; – Provides sparse solutions and supports regularization.	– Computationally and memory-intensive; – Sensitive to kernel and parameter choices; – Primarily used for two-class problems; – No probabilistic output; – Not suitable for large datasets with many features or missing values.
XGBoost	– High performance and scalability; – Customizable with many hyperparameters; – Handles missing values well; – Provides feature importance for interpretability.	– Computationally and memory-intensive; – Prone to overfitting, especially on small datasets; – Hyperparameter tuning is time-consuming.
Artificial Neural Networks (ANN)	– Capable of parallel processing; – Can store and process data even with missing pairs; – Gradual degradation rather than sudden failure; – Learns from past events for decision-making.	– Dependent on hardware supporting parallel processing; – Complex structure difficult to determine; – Requires numeric data, challenging to understand problem statements; – Reliability issues in solution explanations.
Multi-Layer Perceptron (MLP)	– Handles nonlinearity well; – Scalable with modern technology; – Learns important features automatically; – Supports parallel processing.	– High computational cost; – Performance reliant on proper training; – Increased parameters and node redundancy.
Convolutional Neural Network (CNN)	– Excels in detecting patterns in images, videos, and audio; – Robust to transformations (translation, rotation, scale); – End-to-end training without manual feature extraction; – Handles large data well with high accuracy.	– Expensive and memory-intensive training; – Prone to overfitting without sufficient data or regularization; – Requires large labelled datasets; – Limited interpretability.

## Data Availability

The data that support the findings of this study are available from the corresponding author upon reasonable request.
